# Peripheral Signatures of Disease Heterogeneity

**DOI:** 10.1002/alz.086479

**Published:** 2025-01-03

**Authors:** Lenora A Higginbotham

**Affiliations:** ^1^ Emory University, Atlanta, GA USA

## Abstract

**Background:**

There is currently an unmet need for novel accessible biomarkers that capture the complex and heterogenous pathophysiology of Alzheimer’s disease (AD). Over the past decade, the systems‐based multi‐omic approaches employed by the Accelerating Medicines Partnership in AD (AMP‐AD) have resulted in the identification of promising peripheral markers of disease heterogeneity. This scientific review will highlight these advances with a particular focus on the consortium’s successes in peripheral protein biomarker discovery in cerebrospinal fluid (CSF) and plasma.

**Methods:**

The AMP‐AD consortium has applied a brain‐to‐biofluids approach to biomarker discovery designed to identify CSF and plasma markers that reflect the heterogeneous corticolimbic pathophysiology of AD. The successful identification of such biomarkers has relied on a combination of innovative multi‐omic techniques, sophisticated data integration strategies, and informative machine learning algorithms. Network‐based proteomic analysis has proven a powerful tool for advancing this brain‐to‐biofluids strategy.

**Results:**

Network‐based proteomic analysis across hundreds of human brain tissues from AMP‐AD institutions has identified highly reproducible alterations in a variety of co‐expressed protein groups or “modules” in AD. These disease‐associated modules reflect diverse pathophysiology, including synaptic, metabolic, vascular, and inflammatory dysfunction. Integrative analyses have demonstrated that these brain‐based alterations are highly reflected in the AD CSF proteome. AMP‐AD researchers recently validated a 48‐marker brain‐derived CSF panel that can reliably predict cognitive trajectory, amyloid / tau status, and the evolution of neuroimaging markers. In a separate analysis, CSF levels of select matrisome heparin‐binding proteins originally identified in brain network studies discriminated autosomal dominant AD mutation carriers years prior to symptom onset as well or even better than traditional amyloid and tau measures. These matrisome markers have also demonstrated reliable disease‐associated alterations in heparin‐enriched plasma, highlighting their potential as AD blood‐based biomarkers. Finally, emerging research suggests the CSF and plasma marker profiles generated from these network‐based analyses can delineate biological subtypes of AD with distinct clinicopathological phenotypes.

**Conclusions:**

Over the past decade, AMP‐AD has successfully leveraged brain‐to‐biofluid multi‐omic approaches to identify and validate promising peripheral biomarkers of AD. Network‐based proteomics has proven a valuable tool in furthering these biomarker efforts and advancing precision medicine within this complex and heterogeneous disease.

